# Pediatric pineoblastoma: A pooled outcome study of North American and Australian therapeutic data

**DOI:** 10.1093/noajnl/vdac056

**Published:** 2022-04-14

**Authors:** Jordan R Hansford, Jie Huang, Raelene Endersby, Andrew J Dodgshun, Bryan K Li, Eugene Hwang, Sarah Leary, Amar Gajjar, Katja Von Hoff, Olivia Wells, Alison Wray, Rishi S Kotecha, David R Raleigh, Schuyler Stoller, Sabine Mueller, Steven E Schild, Pratiti Bandopadhayay, Maryam Fouladi, Eric Bouffet, Annie Huang, Arzu Onar-Thomas, Nicholas G Gottardo

**Affiliations:** Children’s Cancer Center, Royal Children’s Hospital, Melbourne, Victoria, Australia; Department of Pediatrics, University of Melbourne, Melbourne, Victoria, Australia; Cell Biology and Cancer Division, Murdoch Children’s Research Institute, Melbourne, Victoria, Australia; Michael Rice Cancer Centre, Women and Children’s Hospital, North Adelaide, South Australia, Australia; South Australia Health and Medical Research Institute, Adelaide, South Australia, Australia; Faculty of Health and Medical Sciences, South Australia Immunogenomics Cancer Institute, University of Adelaide, Adelaide, South Australia, Australia; Department of Biostatistics, St. Jude Children’s Research Hospital, Memphis, Tennessee, USA; Brain Tumor Research Program, Telethon Kids Cancer Centre, Telethon Kids Institute, University of Western Australia, Perth, Western Australia, Australia; Children’s Hematology/Oncology Center, Christchurch Hospital, Christchurch, New Zealand; Division of Hematology/Oncology, Cell Biology Research Program, Arthur and Sonia Labatt Brain Tumor Research Institute, The Hospital for Sick Children, Toronto, Ontario, Canada; Department of Pediatrics, Medical Biophysics, Lab Medicine and Pathobiology University of Toronto, Toronto, Ontario, Canada; Division of Oncology, Children’s National Hospital, Washington, DC, USA; Seattle Children’s Hospital, Seattle, Washington, USA; University of Washington, Seattle, Washington, USA; Fred Hutchinson Cancer Research Center, Seattle, Washington, USA; St Jude Children’s Research Hospital, Memphis, Tennessee, USA; Department of Pediatric Hematology and Oncology, Charité-Universitätsmedizin Berlin, Berlin, Germany; Freie Universität Berlin, Berlin, Germany; Humboldt Universität zu Berlin, Berlin, Germany; Children’s Cancer Center, Royal Children’s Hospital, Melbourne, Victoria, Australia; Children’s Cancer Center, Royal Children’s Hospital, Melbourne, Victoria, Australia; Department of Pediatrics, University of Melbourne, Melbourne, Victoria, Australia; Cell Biology and Cancer Division, Murdoch Children’s Research Institute, Melbourne, Victoria, Australia; Department of Neurosurgery, Royal Children’s Hospital, Melbourne, Victoria, Australia; Department of Clinical Hematology, Oncology, Blood and Marrow Transplantation, Perth Children’s Hospital, Perth, Western Australia, Australia; Telethon Kids Cancer Centre, Telethon Kids Institute, University of Western Australia, Perth, Western Australia, Australia; Curtin Medical School, Curtin University, Perth, Western Australia, Australia; Departments of Radiation Oncology and Neurological Surgery, University of California San Francisco, San Francisco, California, Australia; Department of Pediatric Oncology, University of California San Francisco, San Francisco, California, Australia; Department of Pediatric Oncology, University of California San Francisco, San Francisco, California, Australia; Department of Radiation Oncology, Mayo Clinic, Phoenix, Arizona, USA; Boston Children’s Hospital, Dana-Farber Children’s Institute, Boston, Massachusetts, USA; Department of Neuro-Oncology, Division of Hematology/Oncology, Nationwide Children’s Hospital, Columbus, Ohio, USA; Department of Pediatrics, The Hospital for Sick Children/University of Toronto, Toronto, Ontario, Canada; Division of Hematology/Oncology, Cell Biology Research Program, Arthur and Sonia Labatt Brain Tumor Research Institute, The Hospital for Sick Children, Toronto, Ontario, Canada; Department of Pediatrics, Medical Biophysics, Lab Medicine and Pathobiology University of Toronto, Toronto, Ontario, Canada; Department of Biostatistics, St. Jude Children’s Research Hospital, Memphis, Tennessee, USA; Brain Tumor Research Program, Telethon Kids Cancer Centre, Telethon Kids Institute, University of Western Australia, Perth, Western Australia, Australia; Division of Paediatrics, School of Medicine, University of Western Australia, Perth, Western Australia, Australia

**Keywords:** pediatrics, pineoblastoma, retrospective study

## Abstract

**Background:**

Pineoblastoma is a rare brain tumor usually diagnosed in children. Given its rarity, no pineoblastoma-specific trials have been conducted. Studies have included pineoblastoma accruing for other embryonal tumors over the past 30 years. These included only occasional children with pineoblastoma, making clinical features difficult to interpret and determinants of outcome difficult to ascertain.

**Patients and Methods:**

Centrally or independently reviewed series with treatment and survival data from North American and Australian cases were pooled. To investigate associations between variables, Fisher’s exact tests, Wilcoxon-Mann-Whitney tests, and Spearman correlations were used. Kaplan-Meier plots, log-rank tests, and Cox proportional hazards models were used in survival analyses.

**Results:**

We describe a pooled cohort of 178 pineoblastoma cases from Children’s Oncology Group (n = 82) and institutional series (n = 96) over 30 years. Children <3 years of age have significantly worse survival compared to older children, with 5-year progression-free survival (PFS) and overall survival (OS) estimates of 13.5 ± 5.1% and 16.2 ± 5.3%, respectively, compared with 60.8 ± 5.6% and 67.3 ± 5.0% for ≥3 years old (both *P* < .0001). Multivariable analysis showed male sex was associated with worse PFS in children <3 years of age (hazard ratio [HR] 3.93, 95% CI 1.80-8.55; *P* = .0006), suggestive of sex-specific risks needing future validation. For children ≥3 years of age, disseminated disease at diagnosis was significantly associated with an inferior 5-year PFS of 39.2 ± 9.7% (HR 2.88, 95% CI 1.52-5.45; *P* = .0012) and 5-year OS of 49.8 ± 9.1% (HR 2.87, 95% CI 1.49-5.53; *P* = .0016).

**Conclusion:**

Given the rarity of this tumor, prospective, collaborative international studies will be vital to improving the long-term survival of these patients.

**Key Points**

- We present the largest pooled outcome study of the pediatric brain tumor pineoblastoma.
- Young children do exceptionally poorly.
- Older children without metastatic disease do markedly better.

**Importance of the Study**

We present the largest pooled case series of pediatric pineoblastoma reported to date. Current radiation and chemotherapy approaches in older children result in good survival rates. In stark contrast to older patients and comparable to previous reports, the survival of young children is dismal regardless of the therapeutic approach. This provides strong rationale for age- and molecular-based risk stratification for future pineoblastoma trials and highlights the need to identify innovative treatments for infants aged <3 years and children ≥3 years of age with metastatic disease that build on the backbone of conventional chemotherapy and radiotherapy.

Pineoblastoma is a rare and aggressive embryonal tumor of the pineal region primarily affecting children.^1^ The rarity of pineoblastoma has resulted in no clinical trials solely for this disease. Historically, due to their histologic similarity to other central nervous system embryonal tumors, these tumors have been treated on trials for high-risk medulloblastoma and the tumor previously termed central nervous system primitive neuroectodermal tumors (CNS-PNET) or off trial using the same therapies.^2–24^ Consequently, older children with pineoblastoma have generally been treated using aggressive resection, high-dose craniospinal irradiation (CSI), and intensive platinum and alkylator-based chemotherapy, while “Baby Brain” approaches avoiding or delaying radiotherapy using intensive chemotherapy regimens including high-dose chemotherapy with autologous stem cell rescue (ASCR) were utilized for infants and young children. Most of these series have included a relatively small number of patients, making meaningful clinical conclusions difficult to ascertain; as such, the optimal treatment for pineoblastoma remains unknown.

To overcome the small patient numbers, groups have sought to pool patient data. The European Society for Pediatric Oncology (SIOP-E) and the US Head Start groups reported their pooled patient analysis of 135 patients with pineoblastoma revealing a 5-year overall survival (OS) of only 12 ± 4% for patients <4 years old compared with 73 ± 7% for patients aged ≥4 years without metastases. This analysis concluded that age, the use of radiation therapy, and metastatic status were prognostic features significantly predictive of outcome.^25^

Recently integrative epi/genomic analyses have revealed that analogous to the vast majority of other CNS tumors, pineoblastoma is biologically heterogeneous and is composed of 5 core distinct molecular disease subgroups with unique clinical features and survival outcomes.^26–29^ These include subgroups driven by defective microRNA biogenesis including alterations in *DICER1*, *DROSHA*, or *DGCR8*, termed PB-miRNA1 and PB-miRNA2. Pineal parenchymal tumors of intermediate differentiation (PPTID) are characterized by hotspot mutations in *KBTBD4*, which encodes an adaptor protein involved in protein degradation. These 3 subgroups are generally seen in older children and associated with more favorable outcomes in the context of radiation therapy (5-year OS 60%-100%). The fourth and fifth pineoblastoma subgroups, called PB-MYC/FOXR2 and PB-RB1 are characterized by an oncogenic MYC-miR-17/92-RB1 axis and are generally seen in infants and young children, precluding utilization of radiation-based strategies, and are linked with dismal outcomes with 5-year OS ranging from 0% to 25%. These reports serve to further highlight the importance of early molecular characterization of rare diseases to assist in medical decision making and to best design and interpret clinical trials. Despite the relatively large patient numbers included in these series, definitive clinical conclusions to assist in patient management are difficult to derive due to limited matched clinical and molecular subgroup data.

To identify a therapeutic backbone on which to build a future collaborative clinical trial, we undertook a pooled analysis of patients diagnosed with pineoblastoma from North America and Australia.

## Methods

Patients were identified through PubMed searches using the search terms “Pineoblastoma, 1993-2018,” “PNET, 1993-2018.” Individual patient-level data collected from Pediatric Oncology Group (POG), Children’s Cancer Group (CCG), and Children’s Oncology Group (COG) front-line clinical trials were obtained with permission from the COG. Where series were identified through series-level data, institutional investigators were contacted to expand the patient-level data including the Hospital for Sick Children, University of California San Francisco, Mayo Clinic, and Dana-Farber Cancer Institute series and the author’s (J.R.H.) own institutional series (Royal Children’s Hospital, Melbourne, Australia). Only studies published in English-describing survival data were included. Only cases that were either histopathologically centrally or independently reviewed by the authors were included. Full-text copies of all case series and individual patient data from the POG, CCG, and COG clinical trials were independently studied by 2 authors (J.R.H. and N.G.G.), who assessed the eligibility of each case based on data quality and extracted data from the text. Cases from Europe, Head Start, and St Jude Children’s Research Hospital series were excluded as they have been reported previously.^24,25^ All data were collected in accordance with the approval of institutional research ethics boards.

**Figure 1.**
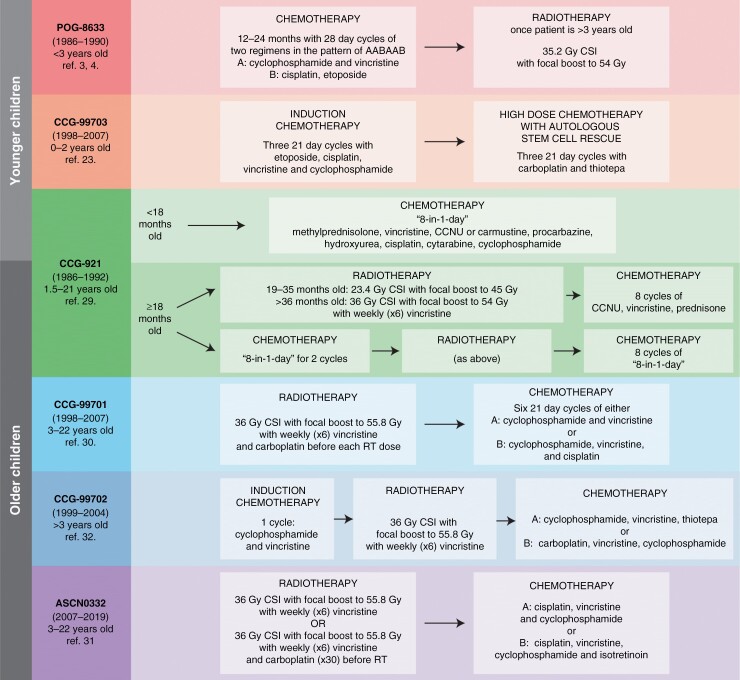
Comparison of clinical trial protocols used in the treatment of children diagnosed with pineoblastoma over the past 4 decades. All protocols involved maximal safe surgical resection followed by chemotherapy ± radiotherapy as shown.

Six prospective clinical trials were included in the cohort including studies POG-8633, CCG-921 CCG-99701, CCG-99702, CCG-99703, and ACNS0332 (see Figure 1 for summary).

### Statistical Methods

Fisher’s exact tests, Wilcoxon-Mann-Whitney (WMW) tests, and Spearman correlations were used to investigate associations between variables. Kaplan-Meier (KM) plots, log-rank tests, and Cox proportional hazards models were used in survival analyses. Multivariable models were constructed using backward elimination where the number of events was adequate to accommodate more than 1 covariate. For progression-free survival (PFS) and OS, the full model included sex, disseminated disease, extent of resection, radiation therapy, and chemotherapy. Variables were eliminated one at a time until all variables remaining in the model were significant. PFS was defined as the time to disease progression, disease relapse, or death by any cause measured from the date of study enrollment. OS was defined as the time to death by any cause measured from the date of study enrollment. Subjects without an event were censored at the time of last follow-up. A significance level of <0.05 was used throughout without adjusting for multiplicity.

## Results

A total of 89 studies and institutional series were identified describing 508 cases. These included 6 studies for which the data were extracted from COG clinical trial databases, 9 published series, and 1 institutional series. After removal of duplicate reports and inclusion of those centrally reviewed cases with sufficient case-specific information particularly treatment and survival data (including some incomplete), 16 unique cohorts reporting 178 cases were included in this analysis (Table 1; Supplementary Figure 1).^13,14,30–35^ Of the 178 cases, 82 (46%) cases were patients from COG trials.

**Table 1.** Distribution of Data Sources

Trials or Papers	Overall		Age Unknown		Age <3 Years		Age ≥3 Years	
	n	%	n	%	n	%	n	%
White, 1993	2	1.1	0	0.0	1	2.0	1	0.8
Schild, 1993	6	3.4	0	0.0	3	6.1	3	2.5
POG8633	11	6.2	0	0.0	11	22.4	0	0.0
CCG921	19	10.7	0	0.0	7	14.3	12	9.8
Prados, 1996	3	1.7	0	0.0	0	0.0	3	2.5
Mikaeloff, 1998^a^	7	3.9	7	100.0	0	0.0	0	0.0
Gilheeney, 2008	11	6.2	0	0.0	2	4.1	9	7.4
Johnston, 2008	9	5.1	0	0.0	5	10.2	4	3.3
Serowka, 2010	1	0.6	0	0.0	1	2.0	0	0.0
CCG99701	25	14.0	0	0.0	0	0.0	25	20.5
CCG99702	3	1.7	0	0.0	0	0.0	3	2.5
CCG99703	8	4.5	0	0.0	8	16.3	0	0.0
RCH	11	6.2	0	0.0	6	12.2	5	4.1
Raleigh_UCSF	19	10.7	0	0.0	2	4.1	17	13.9
ACNS0332	27	15.2	0	0.0	1	2.0	26	21.3
HSC	16	9.0	0	0.0	2	4.1	14	11.5
Total	178	100.0	7	100.0	49	100.0	122	100.0

Abbreviations: CCG, Children’s Cancer Group; COG, Children’s Oncology Group; HSC, Hospital for Sick Children; POG, Pediatric Oncology Group; RCH, Royal Children’s Hospital; UCSF, University of California, San Francisco.

^a^Seven patients in study but age unknown; Data listed from oldest to more contemporary cohorts.

Table 2 demonstrates variables of interest for this study which included: age at diagnosis (years), sex (female/male), disseminated disease (M0/M+), extent of resection (gross total resection (GTR)/non-GTR), radiation therapy received at any time point (yes/no), radiation therapy focal boost (yes/no), radiation therapy focal dose (Gy), CSI (yes/no), CSI dose (Gy), where data were available, chemotherapy (yes/no), and high-dose chemotherapy with ASCR (yes/no). Seven patients were excluded from this analysis as age was not available.

**Table 2.** Characteristics of 171 Included Pineoblastoma Patients Separated by Age Cohort

	Age <3 Years		Age ≥3 Years	
	n	%	n	%
Sex				
Female	20	40.8	61	50
Male	23	46.9	56	45.9
Unknown	6	12.2	5	4.1
Disseminated disease				
M0	28	57.1	78	63.9
M+	16	32.7	36	29.5
Unknown	5	10.2	8	6.6
Gross total resection				
GTR	6	12.2	42	34.4
Non-GTR	39	79.6	73	59.8
Unknown	4	8.2	7	5.7
Radiation therapy				
No	22	44.9	1	0.8
Yes	18	36.7	112	91.8
Unknown	9	18.4	9	7.4
Focal boost				
No	1	2	3	2.5
Yes	16	32.7	48	39.3
Unknown	32	65.3	71	58.2
CSI				
No	2	4.1	5	4.1
Yes	14	28.6	53	43.4
Unknown	33	67.3	64	52.5
Chemotherapy				
No	7	14.3	5	4.1
Yes	40	81.6	113	92.6
Unknown	2	4.1	4	3.3
High-dose chemotherapy with autologous stem cell rescue				
No	22	44.9	73	59.8
Yes	12	24.5	18	14.8
Unknown	15	30.6	31	25.4
Total	49	100	122	100

Abbreviations: CSI, craniospinal irradiation; GTR, gross total resection.

The definition of infant and young child varies globally. In North America and Australia, an infant is generally defined as <3 years of age. Of 178 patients, 49 (27.5%) patients were <3 years of age. Age stratified analyses showed a male to female ratio of 1.15:1 for those aged <3 years of age and 0.92:1 for ≥3 years of age. The cohort with dissemination at presentation occurred in 52 patients (29.2%), of which 16 (16/49 [32%]) were <3 years of age and 36 (36/129 [28%]) were ≥3 years of age. Of 178 patients there was no significant association between age and the presence of disseminated disease (*P* = .9513). There was a significant association between age and extent of resection; compared to patients with GTR, on average, patients with non-GTR were younger at diagnosis (median age 4.6 years for non-GTR vs 9.2 years old for GTR; WMW *P* = .0009). As expected, younger age at diagnosis was significantly associated with not receiving radiation therapy (median age no radiation vs radiation therapy was 1.1 and 8.5 years old, WMW *P* < .0001). Younger age was also associated with receiving a lower focal dose of radiation therapy (data not shown). Also not surprisingly, being treated with radiation and not receiving high-dose chemotherapy with ASCR were associated with each other as many infant clinical trial treatment protocols utilize high-dose chemotherapy with ASCR in place of radiation therapy. This was not relevant for children older than 3. Apart from 1 patient, all children ≥3 received radiation therapy. Age was not associated with other variables.

**Figure 2.**
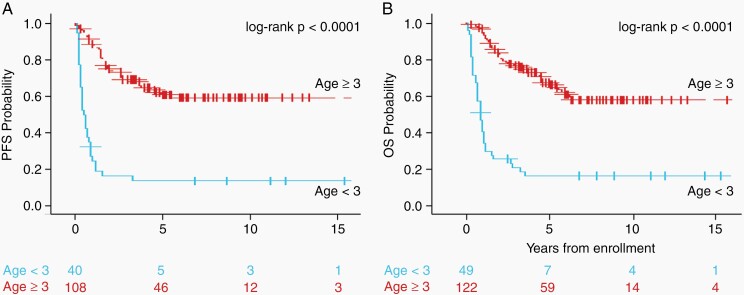
Kaplan-Meier curves showing (A) PFS and (B) OS for children with pineoblastoma according to age at diagnosis (<3 years, *blue*; ≥3 years, *red*). Abbreviations: OS, overall survival; PFS, progression-free survival.

Given patient age played a major contribution in treatment assignment, we analyzed survival outcomes in 2 separate age-based cohorts: <3 years and ≥3 years (Figure 2). Figure 2 shows KM curves for PFS and OS for patients <3 years old at diagnosis compared with those ≥3 years of age at diagnosis. For the PFS analysis, 40 patients were included in the <3 years cohort and 34 of these patients progressed with a median follow-up of 9.9 years (range 0.8-15.3 years). There were 108 patients in the ≥3 years cohort, of which 40 progressed with a median follow-up of 6.2 years (range 0.3-36.3 years). The 5-year PFS estimates were 13.5 ± 5.1% for patients <3 years old, whereas patients ≥3 years old had 5-year PFS estimates of 60.8 ± 5.6% (log-rank *P* < .0001). Median time to progression was 4.8 months in the <3 years cohort and 21 months in the ≥3 years cohort. Forty-nine patients were included in the OS analyses in the <3 years old and 122 patients contributed to the analysis for children ≥3 years old. There were 40 deaths (81.6%) in the <3 years old cohort and 43 deaths (35.3%) in the ≥3 years old cohort. The 5-year OS estimates were 16.2 ± 5.3% for patients in the <3 years old cohort (median follow-up of 8.9 years [range 0.8-15.3 years]), compared with 67.3 ± 5.0% for patients in the ≥3 years old cohort (log-rank *P* < .0001) (median follow-up of 6.1 years [range 0.3-36.3 years]).

### Less Than 3 Years of Age at Diagnosis Cohort

**Figure 3.**
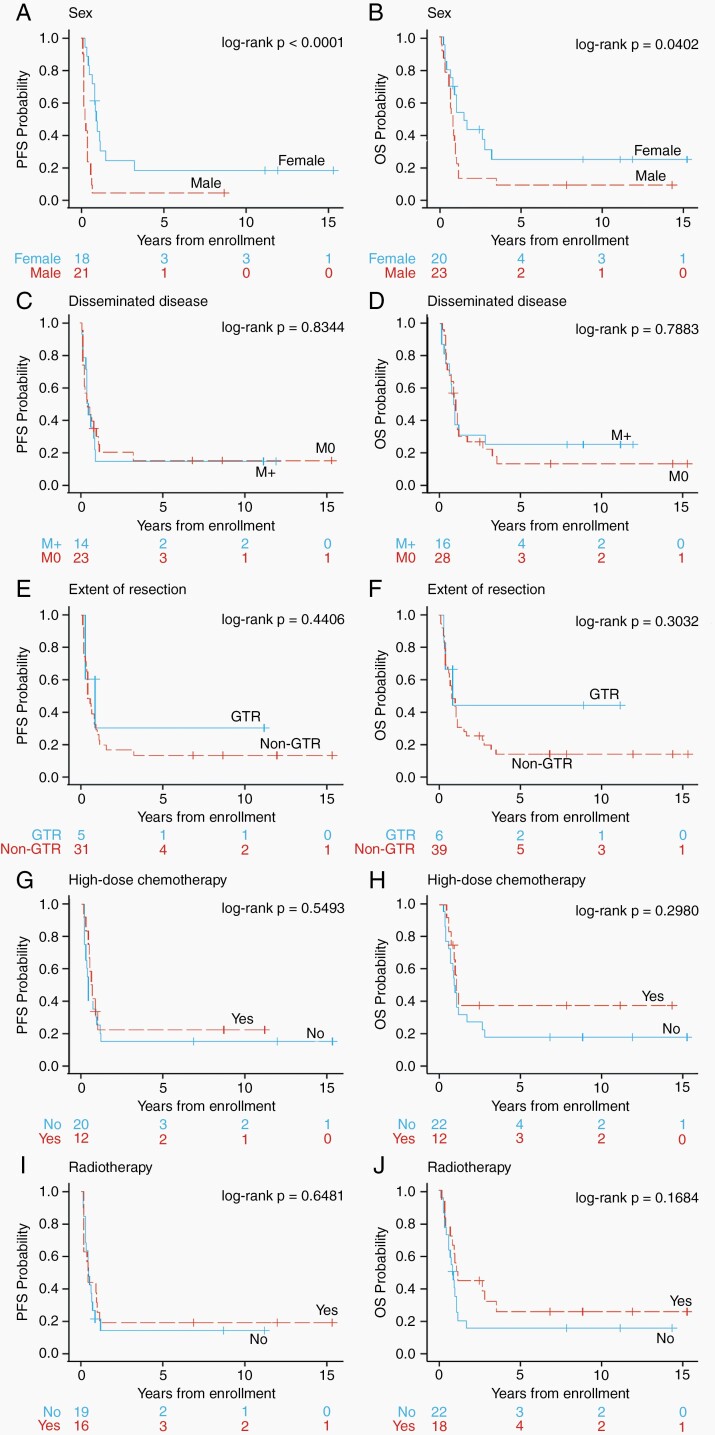
Kaplan-Meier univariable curves for patients aged <3 years at diagnosis of pineoblastoma showing PFS (*left*) and OS (*right*). Factors compared were (A, B) sex, (C, D) metastatic disease at presentation, (E, F) extent of resection, (G, H) chemotherapy, (I, J) high-dose chemotherapy (with ASCR), or (K, L) radiotherapy. Abbreviations: ASCR, autologous stem cell rescue; OS, overall survival; PFS, progression-free survival.

For the <3 years of age cohort, KM plots for PFS and OS comparing variables are shown in Figure 3. Based on log-rank test, the univariate results show that male patients had worse PFS (*P* < .0001) and OS (*P* = .0402) than did female patients (Figure 3A and B). Neither disseminated disease nor extent of resection were significantly associated with PFS or OS outcomes (Figure 3C–F). Only 3 patients included in the PFS analysis did not receive any chemotherapy preventing a meaningful assessment of the role of chemotherapy in this age group, as such we compared patients who received standard or high-dose chemotherapy with ASCR. Twelve patients received high-dose chemotherapy with ASCR compared to 20 that did not (Table 2). This did not significantly affect either PFS or OS (Figure 3G and H). There were no significant differences in PFS or OS between the 18 patients who received radiation therapy compared to 22 that did not (Figure 3I and J). Unfortunately, insufficient data describing the timing of radiation therapy (ie, up-front or at relapse) or whether treatment was focal, or CSI were available for assessment.

While it is not possible to formally compare clinical trial outcomes, Supplementary Figure 2A and B provides the PFS and OS data for patients in the <3 years of age cohort across the different POG/CCG/COG clinical trials examined. The 5-year PFS estimates for patients treated on CCG-921, CCG-99703, and POG-8633 were 28.6 ± 13.9%, 25.0 ± 12.5%, and 0%, respectively. Patients treated on CCG-921 and CCG-99703 had better 5-year PFS than patients treated on POG-8633 (*P* = .013). Of note, there were no statistically significant differences in OS across the different trials (*P* = .208).

A limitation of retrospective analyses are confounders. For example, patients who had a subtotal resection may have received intensified therapy (high-dose chemotherapy with ASCR or radiation therapy). As such, in addition to KM analysis, we also performed multivariable analysis as described in the methods. This confirmed that in children <3 years of age male sex was associated with worse PFS (hazard ratio [HR] 3.93, 95% CI 1.80-8.55; *P* = .0006). Based on Fisher’s exact test using the entire cohort, there was no association between sex (female/male) and disseminated disease (M0/M+) (*P* = .3912). Where data were available, we also tested for associations between the following pairs of variables in the <3 years of age cohort: disseminated disease vs extent of resection, disseminated disease vs radiation therapy, disseminated disease vs focal boost, disseminated disease vs CSI, disseminated disease vs chemotherapy, disseminated disease vs high-dose chemotherapy, extent of resection vs radiation therapy, extent of resection vs focal boost, extent of resection vs CSI, extent of resection vs chemotherapy, and extent of resection vs high-dose chemotherapy. Based on Fisher’s exact test, no association between any of the pairs of variables tested above was identified.

Overall, patients in the <3 years of age cohort had very poor survival; however, there were 7 out of 49 patients (14%) who survived greater than 5 years. We undertook a detailed analysis of these cases to look for any common themes that may underlie their outcomes (Supplementary Table 1). Two of the 7 long-term survivors experienced progressive disease, but all were alive at last follow-up (median 10.9 years, range: 6.8-15.3 years). Four of the 7 had disseminated disease including the 2 patients who progressed. All patients had surgery, though GTR was only achieved in 2 (29%). All 7 patients were treated with chemotherapy and, of these, 3 patients (2 of which had disseminated disease) received high-dose chemotherapy with ASCR as part of the CCG-99703 protocol. Four of the 7 received CSI (including the 2 patients who had disseminated disease). Of note, none of the 3 patients who received high-dose chemotherapy with ASCR had radiation therapy documented.

### Greater Than 3 Years of Age at Diagnosis Cohort

**Figure 4.**
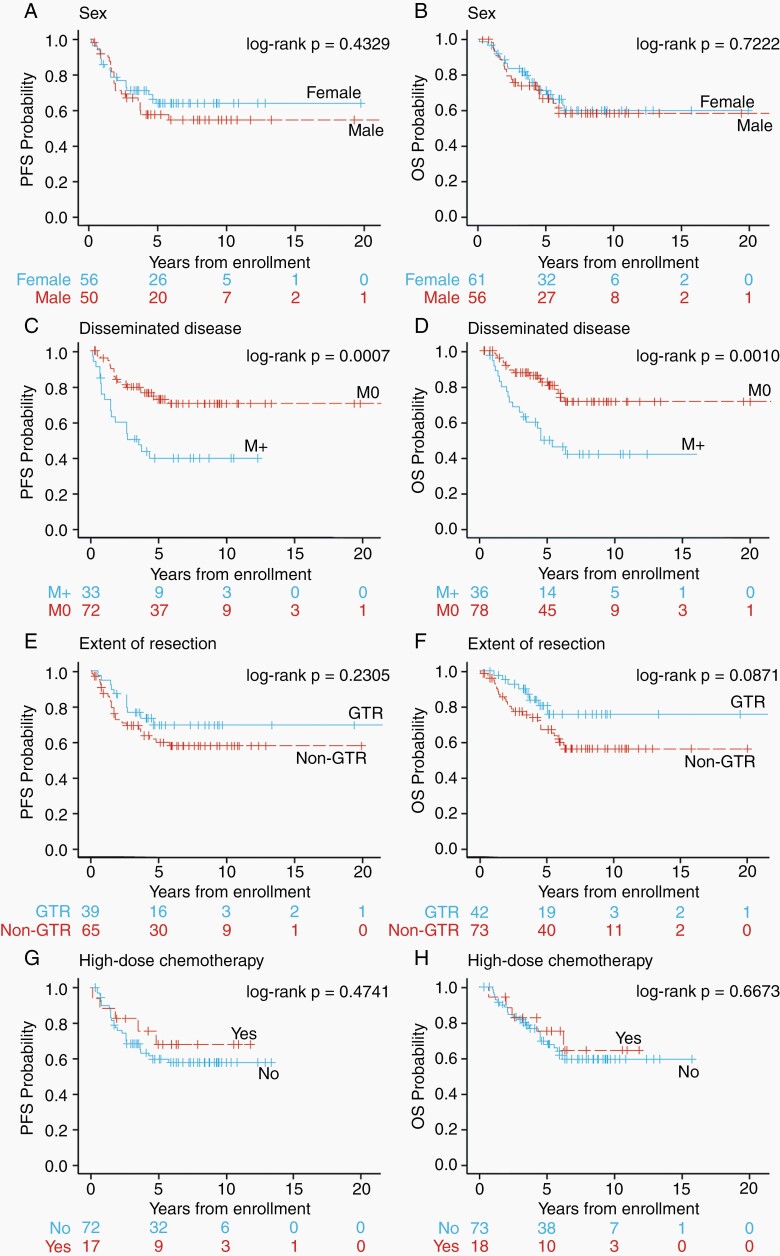
Kaplan-Meier curves showing PFS (*left*) and OS (*right*) for patients aged ≥3 years at diagnosis according to (A, B) sex, (C, D) metastatic disease at presentation, (E, F) extent of resection, or (G, H) high-dose chemotherapy (with ASCR). Abbreviations: ASCR, autologous stem cell rescue; OS, overall survival; PFS, progression-free survival.

For the ≥3 years of age cohort, KM analyses assessing sex, disseminated disease, extent of resection, and treatments are shown in Figure 4. The KM curves reveal that there was no association between PFS or OS with sex (Figure 4A and B). Patients with non-metastatic disease had significantly better 5-year PFS and OS estimates compared to patients with metastatic disease (72.4 ± 6.2% and 82.5 ± 5.1%, for non-metastatic disease vs 39.2 ± 9.7% and 49.8 ± 9.1%, for metastatic disease [*P* = .0007 and .0010, respectively]) (Figure 4C and D). Multivariable analysis revealed that the presence of disseminated disease at diagnosis was significantly associated with an inferior PFS (*P* = .0012; HR 2.88, 95% CI 1.52-5.45) and OS (*P* = .0016; HR 2.87, 95% CI 1.49-5.53). Forty-two patients had a GTR compared with 73 who did not (Table 2). GTR was not associated with differences in either PFS or OS (Figure 4E and F). All but 1 patient with data available received radiation therapy. Due to incomplete descriptions regarding radiation therapy, the contribution of focal radiation therapy only vs CSI approaches to outcome could not be assessed. Eighteen patients received high-dose chemotherapy with ASCR compared with 53 that did not. High-dose chemotherapy was not associated with differences in either PFS or OS (Figure 4G and H).

As was done for infants, KM plots were generated for this age group to explore any differences in PFS and OS outcomes for children treated in the different COG clinical trials (CCG-921, COG-99701, and ACNS0332) but none was observed (Supplementary Figure 2C and D). The 5-year PFS outcomes for patients treated on CCG-921, COG-99701, and ACNS0332, where treatment involved 36 Gy CSI in the majority, were 66.7 ± 12.8%, 58.5 ± 10.5%, and 68.2 ± 10.7%, respectively, while the 5-year OS was 75.0 ± 11.9%, 75.6 ± 9.1%, and 74.6 ± 10.0%, respectively.

## Discussion

To date, no clinical trials have been conducted specific to pediatric pineoblastoma and previous limited series have shown highly variable survival outcomes ranging from 0% to 92% (2-24). Pooled cohort studies can provide useful insights, particularly for very rare disease cohorts.^36^ This current analysis has pooled patient-level data observed over the past 3 decades from North America and Australia and notably included data from subjects treated in 6 prospective COG clinical trials. Another strength of our assessment is the inclusion of only studies where central or independent histopathological review was performed. A major aim of this pooled analysis was to identify a possible therapeutic backbone on which to build a future clinical trial.

Our results are consistent with a previous pooled analysis from the European and Head Start groups,^25^ which had no overlapping patients with our analysis and confirmed the dismal survival for infants, with a 5-year OS of only 16.2 ± 5.3% for children <3 years of age at diagnosis. In contrast, children ≥3 years of age at diagnosis who received multimodal chemotherapy and radiation therapy (which included 36 Gy CSI for the majority) had significantly improved survival, with a 5-year OS of 67.3 ± 5.0%, which increased to 82.5 ± 5.1% for patients with localized pineoblastoma. Of note, Liu et al recently reported a separate series of pineoblastoma patients treated at St Jude Children’s Research Hospital, describing 5-year PFS and OS as 100% for the 20 patients with localized disease treated with 23.4 Gy CSI.^26^

A novel finding from our analysis was the observation that for patients aged <3 years, male sex was associated with significantly inferior PFS compared to females. Male sex is associated with an inferior prognosis in other diseases including medulloblastoma.^37^ Consequently, given the identification of distinct molecular subgroups of pineoblastoma, we speculate that the sex-specific discrepancy observed for children with pineoblastoma may also be due to a greater preponderance of high-risk molecular features in male infants. Consistent with this, a recent meta-analysis of studies investigating the molecular features of pineoblastoma identified a 3.3:1 preponderance of males to females in the poor survival PB-MYC/FOXR2 group, where the median age of diagnosis is 1.4 years.^29^ Notably, in our analysis sex was not associated with prognosis in the older cohort.

For the cohort of patients ≥3 years of age, where generally treatment included surgery, chemotherapy, and radiation therapy, our study clearly demonstrates improved survival compared with those aged <3 years. Consistent with Mynarek et al,^25^ we identified that the presence of metastatic disease at diagnosis was an independent poor prognostic marker in older children, with only 39.2 ± 9.7% and 49.8 ± 9.1% for 5-year PFS and OS for these patients. Although we independently assessed the effects of surgical resection, chemotherapy, and radiation therapy, the role of each modality in contributing to improved outcomes remains unclear and an optimal treatment protocol was not able to be identified. Surprisingly, unlike medulloblastoma, GTR was not associated with an improved outcome. This is similar to recent findings investigating other embryonal tumors with metastatic disease^38^; however, this result must be interpreted carefully given it was from univariate analysis and there is the possibility that the intensity of therapy may have differed for those patients who underwent a STR compared with a GTR. Future work might consider answering this question clearly. Importantly, no difference in PFS or OS was detected among the three different COG studies that enrolled pineoblastoma patients older than 3 years of age, despite the differences in treatment protocols (Figure 1).

The dismal survival of infants (<3 years of age), and the failure to identify a clear therapeutic modality that is superior, highlights new therapeutic approaches are required for this ultra-high-risk group. However, potential themes emerged to consider when developing a treatment backbone to add new therapeutic approaches. Firstly, one of the most striking observations was that of the 7 long-term survivors in this age group, 4 of them had disseminated disease. In contrast to patients ≥3 years of age, disseminated disease was not associated with prognosis. Interestingly, a similar result has also been reported for patients <3 years of age with atypical teratoid rhabdoid tumors.^39^ These observations may be a reflection that the dismal outcome for the <3 years of age group overall negates any underlying prognostic features.

Secondly, for infants aged <3 years, a GTR was only achieved in a minority of patients (6 of 45) (13%) compared with 42 of 115 (36.5%) for ≥3 years of age, possibly indicating the tumors did not lend themselves to a GTR, due to tumor size and location. Resection of pineoblastoma is challenging due to their often hemorrhagic nature (coupled with the small blood volume of the failure to thrive infant), and the pineal relationship to the deep cerebral veins, midbrain, and diencephalon. Additional surgical challenges exist for those <2 years of age, where options to fix the head in an ideal position are limited due to bone thickness and open sutures. Importantly, in the presence of disseminated disease neurosurgeons may also not pursue GTR to reduce the risk of poor neurologic sequelae. In other childhood CNS tumors, most notably medulloblastoma and ependymoma, achieving a GTR or near-total resection has previously been shown to be a critical prognostic variable, and extent of resection plays a major role in defining the subsequent treatment of patients. Until further prospective studies can examine the role of GTR or near-total resection, we continue to advocate for maximal safe resection for children with pineoblastoma.

Finally, while there appeared to be no significant survival benefit for infants who did receive radiation therapy, it should be noted that 4 of the 7 long-term survivors we describe (defined as living more than 5 years from diagnosis) received radiation therapy. Moreover, we confirmed this was first-line CSI in 3 of them, though was undefined in records for the fourth. Also, 2 of the 3 patients who received high-dose chemotherapy with ASCR on the CCG-99703 study, had shorter PFS than OS indicating they likely received salvage therapy, although this was not confirmed.^23^ Radiotherapy is an effective therapy for many CNS tumors, especially embryonal tumors, where it forms the backbone of therapy. However, radiation-sparing or delaying strategies have dominated the management of infants with CNS tumors for several decades given the known negative impacts it has on the developing brain. To this end, a major unresolved question that remains for pineoblastoma is: do infants and young children with pineoblastoma have a dismal prognosis because of the underlying intrinsic aggressive biological nature of their tumors or because radiotherapy is omitted as a therapeutic modality for these patients on account of the unacceptable toxicity, or a combination of both? Given these observations combined with the poor survival in the <3 years of age cohort, inclusion of radiation therapy in future prospective studies should be examined. With the advent of proton radiotherapy with tighter radiation fields, balancing risk vs benefit, future studies should consider including focal radiation in prospective therapeutic trials. Indeed, Mynarek et al noted that 3 of 16 patients <4 years old treated with CSI and a boost, and 3 of 5 treated with high-dose chemotherapy and local radiotherapy survived, prompting the authors to conclude that for patients <4 years old a combination of high-dose chemotherapy and local radiotherapy may be warranted.^25^ Moreover, since the majority of infants relapsed early (median time to progression was 4.8 months in infants vs 21 months in older children) the application of radiation therapy early in treatment deserves consideration. In support of proton-based focal radiation therapy, Jazmati et al retrospectively reviewed proton radiotherapy in 51 infants^40^ (median age of 19 months [range 11-23 months]) with brain tumors, including 1 child with pineoblastoma.^40^ Almost all patients received only focal proton radiotherapy (four received CSI as well). The investigators revealed that it was feasible to deliver this modality to this very young and challenging cohort of patients. The potential late effects remain a concern and prospective studies are needed to confirm the overall benefit of such an approach. However, a study using focal photon-based conformal radiotherapy (CRT) in children and young adults with ependymoma, which included 48 patients <3 years of age (18 were aged 12-18 months old), assessed a comprehensive battery of neurocognitive measures over a 24-month period. Encouragingly, while patients <3 years had significantly lower intelligence quotient (IQ) measures at the start of CRT (89.7 ± 2.2 vs 98.7 ± 3.1 *P* = .034), the IQ of patients <3 years improved over time. Overall, the study revealed stable mean scores on all neurocognitive outcomes. Notably, there was no significant difference between scores for supratentorial compared with infratentorial tumors.^41^

Our analysis did not reveal a clear signal in support of using high-dose chemotherapy with ASCR, as although 3 of the 7 long-term survivors received high-dose chemotherapy as part of the CCG-99703 protocol, 2 of these patients progressed. However, survival analysis comparing the different clinical trials (POG-8633, CCG-921, CCG-99703) revealed a better PFS outcome (*P* = .013) for those patients in the CCG-921 and CCG-99703 studies compared to those patients treated on the less intense POG-8633.

There are several limitations that need to be considered when interpreting retrospective studies including this one. Despite this being the largest report on pineoblastoma, the sample size is still relatively small. This is especially evident after data were divided into the two age-based subgroups. A major limitation of retrospective analyses is the inability to predefine inclusion criteria. The absence of any molecular data for our analysis is a significant weakness given the recent reports outlining the prognostic significance of the different molecular subgroups of pineoblastoma. Furthermore, various data elements of interest were missing or unable to be accessed for some subjects, introducing selection and confounding bias. In this study, some important variables, such as PFS or timing of radiotherapy were not available for several cases. Also, in the majority of North American and Australian centers, radiation-sparing approaches are used in infancy. Thus, it is difficult to comment on the effect of focal vs CSI radiation therapy in this age group. Our inability to determine a physician’s intent also poses challenges in data interpretation, for example, for the small number of patients who did not receive chemotherapy, this may have been due to a decision to follow a palliative course of therapy.

In summary, the outcome of young children is starkly different compared to older patients with a dismal outcome regardless of therapeutic approach. This strongly supports the need for age- and molecular-based risk stratification for future pineoblastoma trials. For infants, the dismal outcome clearly points to the need to identify novel therapeutic approaches. A curative therapeutic modality was not identified here, possibly due to disease molecular heterogeneity among the patients assessed, and our pooled analysis did not uncover a clear therapeutic backbone upon which a future clinical trial should be designed. However, our study provides some important insights. Achieving a GTR is clearly challenging in this cohort, especially for children <3 years of age and maximal safe resection should remain the gold standard. The backbone of therapy should contain an intensive chemotherapy regimen with consideration for high-dose chemotherapy with ASCR, such as CCG-99703. The inclusion of radiation therapy in future studies should be examined and could be limited to focal radiation therapy using protons delivered early given the propensity for early progression.

For children ≥3 years, current radiation and chemotherapy approaches result in good long-term survival rates for patients with localized pineoblastoma, with 5-year PFS and OS of 72.4 ± 6.2% and 82.5 ± 5.1%. However, innovative treatments are required for patients with metastatic disease at presentation to build on the backbone of current conventional radio-chemotherapy strategies such as ACNS0332.

## Supplementary Material

vdac056_suppl_Supplementary_Figure_S1

vdac056_suppl_Supplementary_Figure_S2

vdac056_suppl_Supplementary_Table
