# Supplementary figures and images for: Pediatric pineoblastoma: A pooled outcome study of North American and Australian therapeutic data
Source: Neurooncol Adv. 2022 Apr 14;4(1):vdac056. doi: 10.1093/noajnl/vdac056 (PMC9154333; doi:10.1093/noajnl/vdac056)

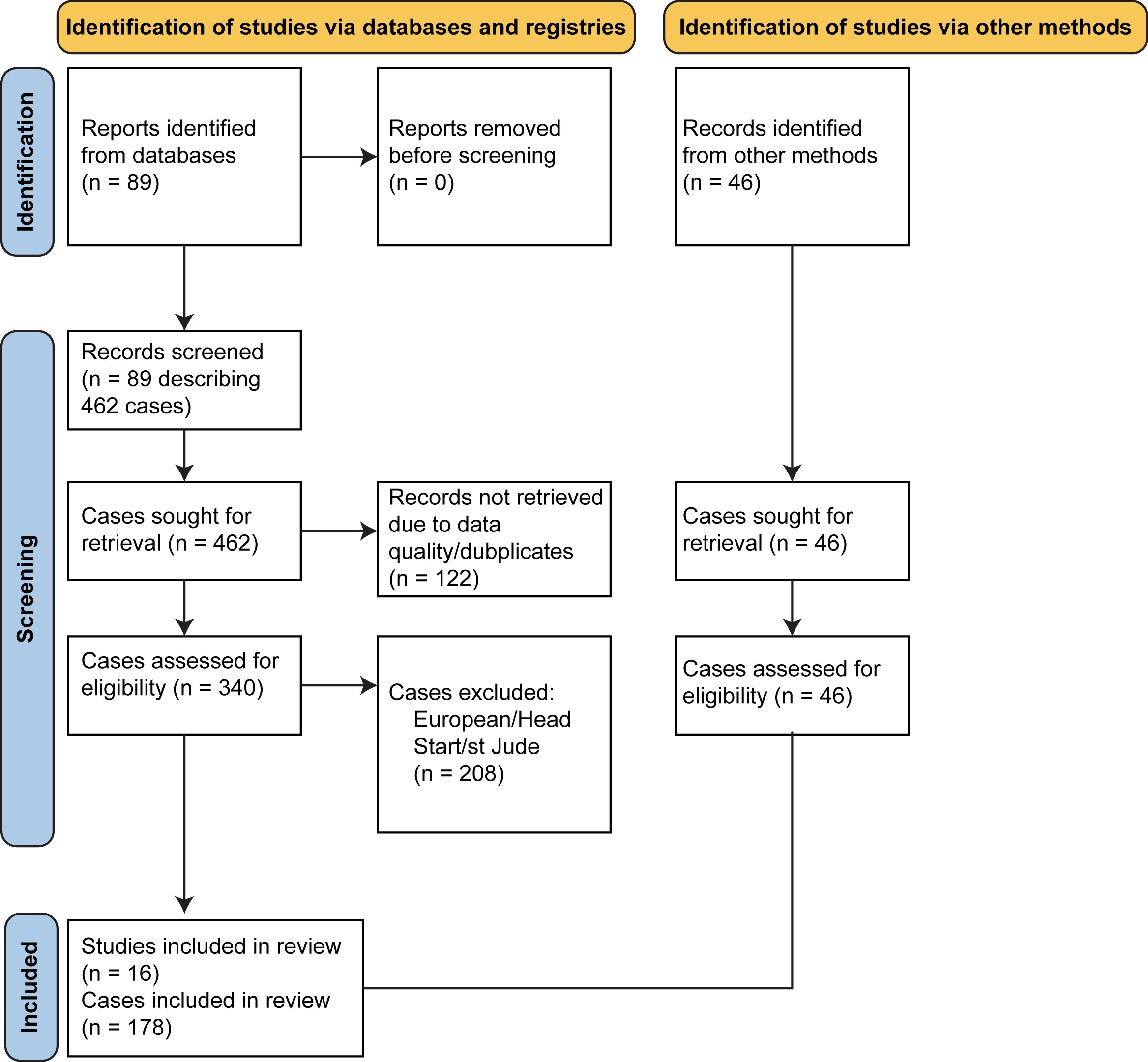

Supplement: vdac056_suppl_Supplementary_Figure_S1 [file vdac056_suppl_supplementary_figure_s1.jpeg]

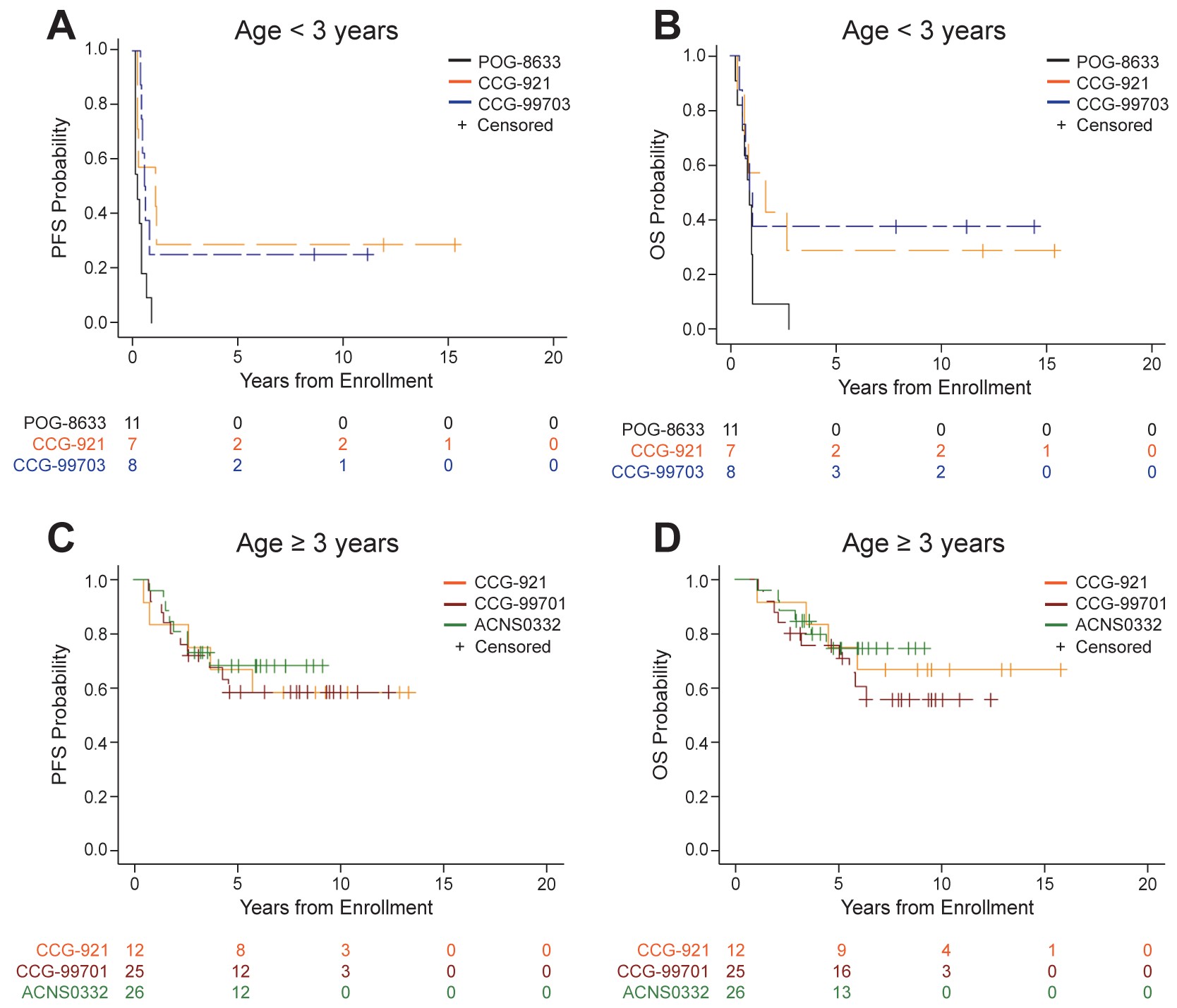

Supplement: vdac056_suppl_Supplementary_Figure_S2 [file vdac056_suppl_supplementary_figure_s2.jpeg]
